# X-Ray Irradiation Induces Oxidative Stress and Upregulates Intestinal Nrf2-Mrp2 Pathway, Leading to Decreased Intestinal Absorption of Valsartan

**DOI:** 10.3390/pharmaceutics17020268

**Published:** 2025-02-17

**Authors:** Yunhua Teng, Jiaojiao Ma, Junxia Zhang, Bohan Liang, Aijie Zhang, Yanjie Li, Shiqi Dong, Huirong Fan

**Affiliations:** 1 Key Laboratory of Radiopharmacokinetics for Innovative Drugs, Institute of Radiation Medicine, Chinese Academy of Medical Sciences and Peking Union Medical College, Tianjin 300192, China; tengyh847@163.com (Y.T.); a1026894769@163.com (B.L.); zhangaijie1986@163.com (A.Z.); 15102274665@163.com (Y.L.); dongsq1314@126.com (S.D.); 2 Graduate School, Tianjin University of Traditional Chinese Medicine, Tianjin 301617, China; 19861402016@163.com (J.M.); jxzhang1204@163.com (J.Z.)

**Keywords:** x-ray irradiation, pharmacokinetics, oxidative stress, valsartan, drug absorption, Mrp2

## Abstract

**Background:** It has been documented that radiation can influence the pharmacokinetics of chemotherapy drugs, yet the underlying mechanisms remain poorly understood. In clinical practice, a considerable number of cancer patients undergo radiotherapy, and those with comorbid hypertension required antihypertensive drugs, including valsartan, an angiotensin II receptor blocker. However, there is no research investigating whether radiotherapy poses a risk of altering the pharmacokinetics. **Objective:** The objective of this study is to investigate the impact of X-ray abdominal irradiation on the pharmacokinetics of valsartan and to preliminarily elucidate the underlying mechanism. **Methods:** The pharmacokinetics of valsartan after X-ray irradiation was investigated in rats and in vitro by detecting the concentration of valsartan in biological samples by LC-MS/MS. The oxidative stress in the intestine and the mRNA expression of partial transporters and Nrf2 in the liver and small intestine were detected by biochemical reagent kit or RT-qPCR. **Results**: In vivo studies showed that X-ray irradiation resulted in a significant decrease in the AUC and C_max_ of valsartan, and the cumulative fractional excretion of valsartan in bile and urine, although there was no significant change in fecal excretion. In vitro studies showed that the uptake of valsartan by both intestine and Caco-2 cells decreased after irradiation, and the cellular uptake could be restored by Mrp2 inhibitor MK571. The levels of GSH, SOD, and CAT in the intestine decreased after irradiation. The mRNA expressions of Mrp2 and P-gp in the intestine or Caco-2 cells were significantly upregulated after irradiation while there was a downregulation of Mrp2 and oatp1b2 in liver. Nrf2 and HO-1 in the intestine were also significantly upregulated, which clarified the involvement of Mrp2 and the possible molecular mechanism. **Conclusions:** Abdominal X-ray irradiation can cause oxidative stress and upregulate intestinal Mrp2, which may be related to oxidative stress and upregulation of Nrf2, reducing intestinal absorption of valsartan and leading to a significant decrease in the blood concentration of valsartan.

## 1. Introduction

In recent years, some studies have found that ionizing radiation, including X-rays and gamma rays, can affect the pharmacokinetics of some drugs in animals [1,2,3]. For example, Hsieh et al. reported that local irradiation could affect the pharmacokinetics of 5-fluorouracil in rats [3], resulting in a decrease in the area under the plasma drug concentration–time curve (AUC) and an increase in the clearance rate (CL) of 5-fluorouracil. In addition, the excretion of the parent drug in the bile increased, whereas there was a decrease observed in the metabolites. Our laboratory has also reported the impact of X-ray irradiation on drug pharmacokinetics in rats, including the reduction in plasma area under the curve (AUC) and excretion of apatinib in rats [4]. Additionally, we observed a decrease in renal excretion of bestatin in rats, resulting in increased body accumulation and blood drug concentration [5]. This phenomenon, in which radiation affects the absorption, distribution, metabolism, or excretion of drugs in the body, leading to changes in the pharmacokinetic characteristics of drugs, is called radiation pharmacokinetics [6] (hereinafter referred to as RT-PK). In clinical practice, the co-administration of radiotherapy and drugs in cancer patients may result in RT-PK, which can potentially alter the pharmacokinetics of the drugs utilized. The occurrence of RT-PK may lead to an elevation in drug exposure or accumulation within the body, thereby potentially inducing more severe toxic side effects. Conversely, it could also result in a reduction in drug efficacy. Consequently, further investigations into the phenomenon of RT-PK and its underlying mechanisms are warranted.

According to the “China Cardiovascular Health and Disease Report 2023” [7], the prevalence of hypertension in China has reached a staggering 245 million individuals. Simultaneously, there exists an overlap between this population and the considerable number of cancer patients in China [8,9]. Firstly, there exist shared risk factors between hypertension and cancer, including advanced age [10], tobacco use [11], diabetes [12], and obesity [13], among others. Secondly, hypertension is a common concomitant disease in cancer patients [11,14,15]. In addition to hypertension associated with certain neoplasms, such as pheochromocytoma [14], secondary hypertension is usually triggered or exacerbated by cancer treatment drugs [16], especially vascular endothelial growth factor inhibitors (VEGFIs) [11,17,18]. For instance, clinical studies have shown that the incidence of hypertension associated with bevacizumab treatment ranges from 26% to 55% [18,19,20]. Inadequate management of hypertension also heightens the risk of cardiotoxicity resulting from cancer treatment [21] and increases the mortality risk associated with cardiovascular disease [9,22,23]. Therefore, it is imperative to enhance both prevention and management strategies for hypertension in individuals with cancer. The rational utilization of antihypertensive medications can effectively assist cancer patients in managing blood pressure and reducing the incidence of cardiovascular events [9,24,25]. Sartans (angiotensin II receptor blockers, ARBs) can reduce blood pressure by blocking the binding of angiotensin II to its receptor [26]. They are the first-line antihypertensive drugs recommended by the European Society of Cardiology [22]. ARBs exhibit the characteristics of a stable antihypertensive effect and few adverse reactions, making them suitable for monotherapy or combination therapy in the prevention and treatment of hypertension, including hypertensive conditions in cancer patients [25]. This raises the question of whether radiation can induce the RT-PK phenomenon in antihypertensive drugs when these patients receive radiotherapy, thereby affecting the effect of blood pressure control and even the life health of cancer patients.

The absorption, distribution, metabolism, and excretion of drugs in the body are significantly influenced by transporters and metabolic enzymes. During the RT-PK phenomenon, alterations in the pharmacokinetic processes of drug absorption, distribution, metabolism, and excretion have been observed [1,2,3,4,5,6]. Previous studies suggest that these changes are likely attributable to the impact of radiation on metabolic enzymes [1] or transporters [5]. However, the mechanism of RT-PK is still poorly understood. Furthermore, ionizing radiation can induce the generation of reactive oxygen species (ROS) [27,28,29,30], leading to oxidative stress [30]. Oxidative stress involves changes in a series of pathways [31,32,33,34,35], such as the antioxidant-related pathway Nrf2-ARE [34], the inflammation-related pathway PI3K [31], etc. Some of these pathways are closely related to the regulation of P-gp [36], Mrp2 [31,32,33,37], and other transporters [36,37]. This suggests the possibility that radiation affects transporter expression through oxidative stress. For instance, nuclear factor erythroid 2-related factor 2 (Nrf2), which plays a crucial role in the body’s regulation of oxidative stress, also exerts transcriptional regulation on multidrug resistance-associated protein 2 (Mrp2) and can be activated by radiation exposure [38]. Our previous study found that abdominal X-ray radiation could affect the pharmacokinetics of various sartans, including valsartan, which was not metabolized in vivo and excreted as the original [39]. Valsartan is a substrate of Mrp2 [40]. Mrp2 is primarily expressed in the small intestine [41], especially in the proximal duodenum and jejunum [42,43], the hepatocyte canalicular membrane [44,45], etc. Mrp2 plays a pivotal role in the efflux of numerous drugs (methotrexate [46], pravastatin [47], and irinotecan [48]), phase II metabolites (acetaminophen sulfate [49] and resveratrol glucuronide/sulfate [50]), and endogenous substances (bilirubin [51] and glutathione S-conjugates [52]) into extracellular fluid (bile, intestinal fluid, and urine) [53]. Consequently, it is closely associated with drug absorption, distribution, and excretion. Therefore, the expression level of Mrp2 may significantly impact valsartan. Additionally, it has been reported that P-glycoprotein (P-gp) can also impact the absorption of valsartan [54,55]. Valsartan is also a substrate for organic anion-transporting polypeptides 1B1/1B3 (OATP1B1/1B3) [40], which are predominantly expressed in the human liver and play a crucial role in drug uptake. Therefore, if ionizing radiation affects the aforementioned transporters, it is highly probable that it will alter the pharmacokinetics of valsartan in vivo.

In summary, the primary objectives of this study were to investigate the impact of X-ray irradiation on the pharmacokinetics of valsartan and elucidate the underlying mechanism, including the alterations in the expression of valsartan-related transporters and the role of oxidative stress and Nrf2 in these changes. The results of this study could provide some reference basis for regulating the medication of valsartan before and after abdominal radiotherapy in cancer patients with hypertension, and even to predict the RT-PK of other drugs.

## 2. Materials and Methods

### 2.1. Chemicals and Regents

Valsartan (CAS:137862-53-4), verapamil (CAS:52-53-9), telmisartan (CAS:144701-48-4), and MK571 (CAS:115104-28-4) were purchased from Macklin (Shanghai, China). Acetonitrile and methanol (HPLC grade) were purchased from Concord Technology (Tianjin, China), sodium carboxymethylcellulose (CMC-Na) and ammonium acetate from Aladdin(Shanghai, China), Dulbecco’s modified eagle medium (DMEM), basal medium, and fetal bovine serum (FBS) from Gibco (Waltham, MA, USA), and penicillin–streptomycin (100×: 10,000 Units) and trypsin from Sigma Aldrich (St. Louis, MO, USA). Triton X-100 and BCA protein concentration assay kits were purchased from Solaibio (Beijing, China), and GSH, SOD, and CAT assay kits were purchased from Nanjing Jianxeng BioEngineering Institute (Nanjing, China). TRIzol reagent was purchased from Invitrogen (Carlsbad, CA, USA).

### 2.2. LC-MS/MS Conditions

Reverse-phase chromatography was performed under gradient conditions. The separation column used was an InfinityLab Poroshell 120 EC-C18 (2.1 × 50 mm, 2.7-Micron) and its operating temperature was 40 °C. The samples were injected into the system through an autosampler at a volume of 1 μL, and the samples were maintained at 4 °C before injection. The mobile phase was composed of (A) water (containing 1 mM ammonium acetate, 0.05% formic acid, and 5% acetonitrile) and (B) acetonitrile. The flow rate was set to 0.3 mL/min, the elution procedure and mobile phase ratio were 0–0.5 min, and 30% B operation was maintained; from 0.5 to 2.0 min, it increased from 30% B to 85% B in 1.5 min; from 2.0 to 4.0 min, running was kept stable at 85% B for 2 min; from 4.0 to 4.1 min, it decreased from 85% B to 30% B within 0.1 min; and from 4.1 to 5.5 min, running was maintained at 30% B.

The analytes were detected and quantified by tandem mass spectrometry (MS/MS) in positive-ion mode. The ion source was electrospray ionization (ESI), and the detection was performed by a triple quadrupole detector. MS parameters were optimized as follows: the ion spray voltage was 5500 V; the ion source temperature was 550 °C; the curtain gas was 50 psi; the entrance potential (EP) was 12 V; the collision cell exit potential (CXP) was 11 V; the collision energy (CE) was 41 V; the declustering potential (DP) was 38 V; the valsartan ion pair was *m/z* 436.1→207.1; and the internal standard (telmisartan) ion pair was *m/z* 515.2→276.1.

### 2.3. Method Validation

A stock solution of 1 mg/mL was obtained by dissolving 10 mg of valsartan/telmisartan standard in 10 mL of methanol and it was stored at –20 ° C. The valsartan stock solution was diluted with pure methanol to obtain a series of gradient concentration calibration curve working solutions and a quality control working solution. The concentrations of the valsartan calibration curve (including the standard concentration 1–7, S1–S7) working solutions were as following: 2, 10, 100, 500, 2500, 4000, and 5000 ng/mL. The concentrations of the lower limit of quantification (LLOQ) and quality control (LQC, MQC, and HQC) working solutions were 2, 5, 1000, and 3750 ng/mL, respectively. The stock solution was diluted to 50 ng/mL with pure methanol to obtain the internal standard working solution.

A total of 50 μL of the prepared valsartan working solution and telmisartan working solution were added to blank rat plasma (50 μL) in turn, and methanol was added as a precipitant to 250 μL. After centrifugation at 16,260× *g* for 10 min, 100 μL of the supernatant was taken for LC-MS/MS analysis. Calibration curve standard samples were also prepared, including double-blank and blank (with IS added) plasma samples.

According to the guidance principles of “M10: Bioanalytical Method Validation and Sample Analysis”, published by ICH (the International Coordinating Committee for Technical Requirements for Medicinal Products for Human Use) [56], combined with the actual requirements for substance quantification in this study, the selectivity, accuracy, precision, and linearity, as well as the extraction recovery and matrix effect of the established LC-MS/MS quantitative analysis method, were validated.

### 2.4. Animals and Treatments

All animal experiments were approved by the Animal Ethics Committee of the Institute of Radiation Medicine, Chinese Medical Sciences. Male Sprague Dawley (SD) rats (weight 200 ± 20 g) were purchased from Beijing Vital River Laboratory Animal Co., LTD. All rats were housed in an SPF animal laboratory (22 ± 2 °C, 40−70% humidity) with an alternating 12 h light–dark cycle. The rats were fed a maintenance diet and had free access to water. All rats were adaptively fed for three days before the experiment and randomly divided into a control group (A) and irradiation group (B). There were 6 rats in group A and B in each study. The control group was not treated before the experiment, and the irradiation group was treated with lead mold to control the irradiated area and received abdominal X-ray irradiation (5 Gy, 2 Gy/min) 24 h before the experiment. The samples collected were processed and then stored at −80 °C until analysis.

#### 2.4.1. In Vivo Pharmacokinetics Studies

Valsartan was administered by gavage (10 mg/kg) after forming a homogeneous suspension (2 mg/mL) with 1% CMC-Na by grinding and stirring. Before and after administration (0.083, 0.25, 0.5, 1, 2, 4, 6, 8, 12, and 24 h), the rats in group A (*n* = 6) and group B (*n* = 6) were anesthetized with isoflurane. Blood samples were collected by the orbital blood sampling method, and whole blood was collected by blood collection tubes coated with heparin sodium. Plasma samples were obtained by centrifugation at 16,260× *g* for 10 min.

#### 2.4.2. In Vivo Excretion Studies

For the VAL urinary and fecal excretion assay, six rats in each group, A and B, were randomly assigned to metabolic cages and administered valsartan (10 mg/kg) via gavage. Feces and urine samples were collected within 12 h before administration and at various time points thereafter. The feces and urine were mixed with a 50% methanol–water solution at a ratio of 1:4, homogenized, and then centrifuged at 16,260× *g* for 10 min. The resulting supernatant was collected as the analytical samples.

For the VAL bile secretion assay, six rats in each group, A and B, underwent bile duct cannulation prior to administration. After anesthesia, the abdominal cavity of the rats was opened, followed by separation and ligation of the distal end of the common bile duct. A small opening was made at the front end of the ligation to insert a catheter, which was then ligated and fixed before suturing the abdominal cavity. Bile was collected through a catheter. The rats were maintained under anesthesia with intraperitoneal injection of normal saline and glucose to ensure stable vital signs. Following surgery, valsartan (10 mg/kg) was administered via gavage, and bile samples were collected at different time points post-administration. The collected bile samples were mixed with 50% methanol–water (ratio 1:9), centrifuged to obtain the supernatant for analysis.

#### 2.4.3. Ex Vivo Inverted Intestinal Sac Absorption Study

Six rats in groups A and B were anesthetized with isoflurane, followed by the opening of the abdominal cavity to separate a 10 cm segment of the jejunum. The distal end of the jejunal segment was ligated in Krebs–Ringer Solution (KRB), and the resulting intestinal sac was turned inside out from the proximal end to form an inverted intestinal sac. Subsequently, 1 mL of KRB buffer at 37 °C was added to the inverted intestinal sac, which was then equilibrated in oxygen-saturated KRB buffer (37 °C) for 5 min. The flipped intestinal sac was transferred to oxygen-saturated KRB buffer (37 °C) containing 10 mM VAL, initiating the timing process. At different time points, 50 μL of solution from the flipped intestinal sac was aspirated and supplemented with an equal volume (50 μL) of blank KRB buffer.

### 2.5. Cell Culture and Treatments

The Caco-2 cells were obtained from Procell Technology (Wuhan, China). The cells were cultured and passaged under controlled conditions of 37 °C, 5% CO_2_, and 95% relative humidity using DMEM supplemented with 10% fetal bovine serum and 1% penicillin–streptomycin. The DMEM was refreshed every alternate day.

#### 2.5.1. Cell Counting Kit-8 Assay

The Caco-2 cells were seeded in 96-well plates at a density of 7000 cells per well. After 48 h of culture, the original medium was removed and replaced with 90 μL of DMEM (without FBS) containing varying concentrations of valsartan (Val, 10 μM), MK571 (10, 50, 100, and 200 μM), or verapamil (Ver, 10, 50, 100, and 200 μM). Following a 6 h incubation period, each well was supplemented with 10 μL of CCK8 reagent and subsequently incubated at 37 °C in the absence of light for an additional 2 h. Subsequently, the absorbance at a wavelength of 405 nm was measured using a microplate reader to determine the cell viability.

#### 2.5.2. Cell Uptake Assay

Caco-2 cells were seeded in 24-well plates at a density of 5 × 10^5^ cells per well. After 14 days of culture, the cells in different plates were divided into the control group and irradiation (IR) group. The cells in the IR group received X-ray irradiation (5 Gy, 2 Gy/min) and recovery for 24 h. The wells were washed three times with PBS (phosphate-buffered saline, prewarmed at 37 °C), and then blank medium or medium containing MK571 (Mrp2 inhibitor, 10 μM) or verapamil (P-gp inhibitor, 100 μM) was added to the wells and preincubated at 37 °C for 5 min. Then, the medium containing valsartan (10 μM) or the medium containing valsartan (10 μM) and inhibitor (10 μM MK571 or 100 μM Ver) was then added, and the timing was initiated, maintaining a constant temperature of 37 °C throughout the experiment. At the indicated time points, the culture medium was removed and each well was rinsed thrice with ice-cold phosphate-buffered saline (PBS). Subsequently, 200 μL of 1% Triton X-100 was added to each well for cellular lysis. The protein concentration in the supernatant was determined by bicinchoninic acid assay (BCA) kit, while the concentration of valsartan was quantified by LC-MS/MS.

### 2.6. Real-Time Quantification Polymerase Chain Reaction (RT-qPCR) Studies

Caco-2 cells, which were cultured to 60% growth density, or SD rats were randomly divided into control group, IR-12 h, IR-24 h, IR-48 h, and IR-72 h irradiation groups (*n* = 4). Except for the control group, all groups received X-ray irradiation at the same dose and using the same method as before. At the indicated time points, the rats were euthanized. The collected jejunum and liver tissues were washed with normal saline and frozen at −80 °C. The Caco-2 cells were removed from the medium and washed three times with PBS. A certain amount of RNA extraction reagent (TRIzol) was added to the aforementioned tissue samples or cells, and the tissue or cells were homogenized. After chloroform was added, the lysate was centrifuged, and the clear supernatant was obtained. RNA was precipitated from the supernatant using isopropanol. The precipitate was washed with 80% ethanol–water to remove impurities, dried, and then re-dissolved in DEPC water to obtain total RNA. The RNA content was determined by measuring the optical absorbance at 260 nm using a NanoDrop 2000 spectrophotometer (Thermo Fisher Scientific, Inc., Waltham, MA, USA). The RNA was reverse transcribed to obtain cDNA templates, which were mixed with the designed primers (Sangon, Inc., Shanghai, China) and TB Green Ex Taq II (Takara, Inc., Kusatsu, Japan) (Table S1) and amplified by real-time fluorescence quantitative PCR (Table S2). β-actin was used as a housekeeping gene, and the relative expression of the target gene was calculated using the 2^−ΔΔCt^ method. The primer sequences are shown in Table S3.

### 2.7. Biochemical Analysis of Oxidative Stress

The rat jejunal tissue (100 mg) was mixed with normal saline at a ratio of 1:9, followed by chopping on ice and homogenization using a homogenizer. Subsequently, the mixture was centrifuged at 3000 rpm and 4 °C for 15 min. The resulting supernatant was utilized to assess the levels of glutathione (GSH), superoxide dismutase (SOD), and catalase (CAT) in intestinal tissue based on the manufacturer’s instructions.

### 2.8. Statistical Analysis

Quantitative analysis of LC-MS/MS results was performed using Analyst 1.6.3 program. The pharmacokinetic parameters were calculated using the WinNonlin 8.3 program. SPSS Statistic 20.0 software was used for statistical analysis. One-way ANOVA was used for comparison among multiple groups, and *p* < 0.05 was considered statistically significant. GraphPad Prism 8.0.2 software was used for generating figures.

## 3. Results

### 3.1. Validation of LC-MS/MS Quantitative Method

As shown in Figure S1, the retention times of valsartan and the internal standard (IS) are 2.11 min and 1.88 min, respectively. The peaks of valsartan and the internal standard are well shaped, while there are no impurity peaks interfering with the retention times of the substances nearby. The baseline response is less than 20% of the valsartan at LLOQ and 5% of the IS, which indicated that endogenous substances in rat plasma do not interfere with the determination of valsartan, and the method has good selectivity and specificity. The results (Table S4) showed that the intra-day and inter-day precision (relative standard deviation, RSD%) of valsartan were between 0.78 and 5.99% and 0.71 and 6.22%, respectively. The accuracy ranged from 100.3 to 104.4%. The intra-day and inter-day precision and accuracy of LLOQ, LQC, MQC, and HQC met the relevant requirements of the M10 guidelines published by the ICH. The calibration curve in rat plasma samples ranged from 2 to 5000 ng/mL. The correlation coefficients of all curves were ≥0.99, indicating that the concentration of valsartan had good linearity in the range of the curve (Table S5).

As shown in Table S6, the matrix effects of LQC and HQC of valsartan were 100.78% and 97.78%, respectively, and the corresponding RSD were 7.96% and 2.58%, respectively, which were within ±15%. The matrix effect of the IS was 103.09%, and the corresponding RSD was 3.24%, which was within ±15%. The extraction recovery rates of LQC and HQC of valsartan were 97.41% and 103.01%, respectively, and the corresponding RSD were 2.96% and 2.60%, respectively, which were within ±15%. The recovery rate of IS was 99.44%, and the corresponding RSD was 2.90%, which was within ±15%. The above results indicate the absence of any co-eluting substance that could impact the ionization of valsartan and the internal standard, while also confirming that the extraction recovery meets quantitative requirements.

### 3.2. Effect of X-Ray Irradiation on the Pharmacokinetics of Valsartan in Rat Plasma

**Figure 1:**
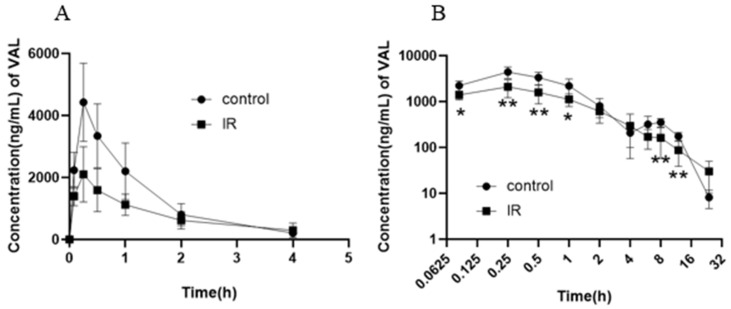
The average blood concentration–time (0–4 h) curve of valsartan (**A**) and logarithmic average blood concentration–time (0–24 h) curve of valsartan (**B**) in rats after abdominal irradiation with 5 Gy of X-rays (Mean ± SD, *n* = 6, * indicates the control group compared, * *p* < 0.05, ** *p* < 0.01).

The LC-MS/MS analysis of plasma samples collected post-drug administration (Figure 1) revealed that valsartan exhibited rapid absorption, with peak plasma concentration observed at approximately 0.25 h, followed by a subsequent faster elimination rate. Interestingly, compared to the control group, the plasma concentration of valsartan in the IR group exhibited a significant decrease during the absorption–distribution phase as well as near its peak (*p* < 0.05). In addition, the plasma concentration of valsartan in the control group increased again from 4 h to 8 h, and the reabsorption of valsartan in the IR group had almost no effect on the plasma concentration. Therefore, the plasma concentration of the IR group was lower than that of the control group during 8–12 h (*p* < 0.05). The pharmacokinetic parameters of valsartan (Table 1) showed that the area under the concentration–time curve (AUC_0–24h_) and maximum plasma concentration (C_max_) of valsartan in the IR group decreased by 37% (*p* < 0.05) and 52% (*p* < 0.05), respectively. The plasma half-life (t_1/2_) of valsartan exhibited an upward trend and was prolonged by 41% (from 3.57 h to 5.03 h); however, the time to peak (T_max_) remained unchanged. These findings suggest that radiation may primarily affect the absorption of valsartan, resulting in a decrease in plasma concentration and overall body exposure.

### 3.3. Effect of X-Ray Irradiation on the Secretion of Bile and Urinary and Fecal Excretion of Valsartan in Rats

**Figure 2:**
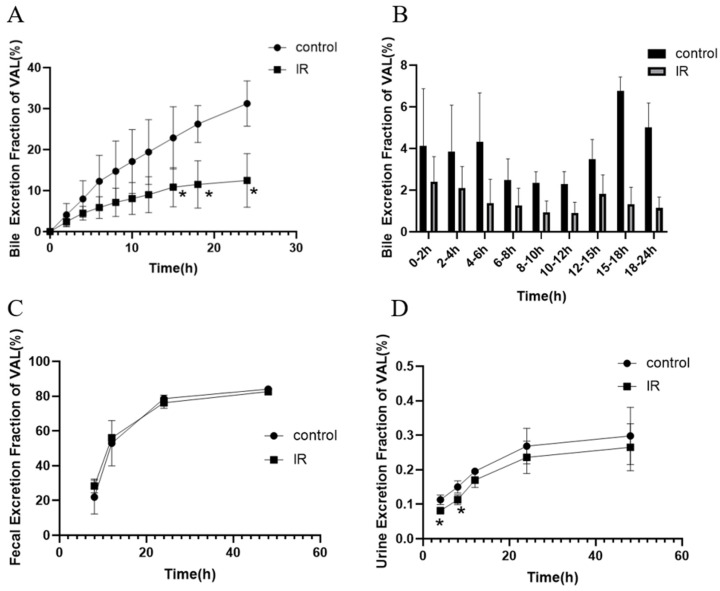
The cumulative excretion fraction of valsartan in bile (**A**), fecal (**C**), urine (**D**), and excretion fraction of valsartan in different time periods in bile (**B**) after abdominal irradiation with 5 Gy of X-rays (Mean ± SD, *n* = 4( **A**,**B**), *n* = 6 (**C**,**D**); * indicates the control group compared, * *p* < 0.05).

The excretion of valsartan was calculated by the concentration and sample volume (or weight), and the fractional excretion of valsartan and the cumulative excretion fraction of valsartan in each time period were obtained through comparison with the corresponding dose administered to the rat. The results showed that the cumulative fractional bile excretion of valsartan in the IR group decreased significantly (Figure 2A), reaching only 10.83%, 11.52%, and 12.67% of the dose at 15 h, 18 h, and 24 h, respectively, which were significantly lower than those in the control group (22.89%, 26.24%, and 31.26%, respectively). The fractional excretion in the IR group was lower than that in the control group (Figure 2B). Indeed, feces were the major route of valsartan excretion in rats, with a cumulative excretion of 83%, but the cumulative fractional excretion of valsartan in feces did not exhibit a statistically significant difference between the IR and control groups (Figure 2C). Valsartan was excreted in a very small amount in the urine (Figure 2D), and the fractional excretion of valsartan was lower in the IR group than in the control group, with a significant difference at 4 h and 8 h. These results indicated that excretion and bile secretion were not responsible for the decrease in plasma concentrations of valsartan.

### 3.4. Effect of X-Ray Irradiation on the In Vitro Absorption of Valsartan

**Figure 3:**
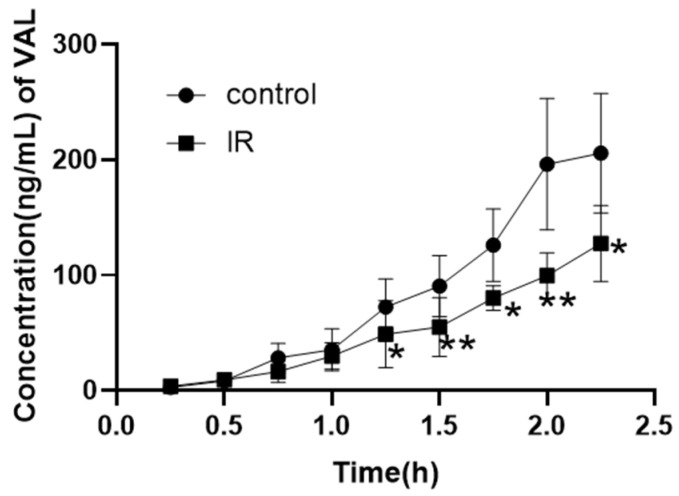
Absorption of valsartan after abdominal irradiation with 5 Gy of X-rays in inverted intestine sac (Mean ± SD, *n* = 6, * indicates the control group compared, * *p* < 0.05, ** *p* < 0.01).

In order to exclude the influence of other factors in vivo, an in vitro inverted intestinal sac assay was designed to explore the role of intestine absorption. The in vitro absorption of the IR group exhibited a significant decrease (Figure 3), which was observed from 1.25 h onwards. The concentration of valsartan in the intestinal sac at the final time point was measured to be 127 ± 32.94 ng/mL in the IR group, demonstrating a significant reduction of 38% compared to that observed in the control group (205.6 ± 51.72 ng/mL). These findings strongly suggest that diminished absorption is primarily responsible for the decline in plasma concentration of valsartan.

### 3.5. Effect of X-Ray Irradiation on the Uptake of Valsartan in Caco-2 Cell

**Figure 4:**
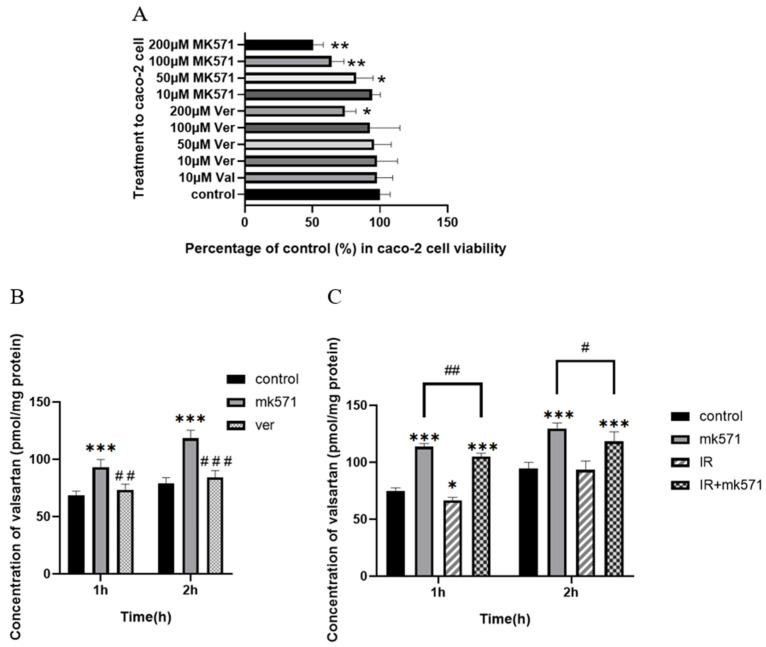
Percentage of Caco-2 viability after treatment with valsartan and inhibitors of Mrp2 and P-gp (**A**), concentration of valsartan in Caco-2 cells after treatment with valsartan and inhibitors of MK571 or Ver (**B**), and concentration of valsartan in Caco-2 cells after irradiation with 5 Gy of X-rays (**C**) (Mean ± SD, *n* = 4, * indicates the control group compared, # indicates the MK571 group compared, * *p* < 0.05, ** *p* < 0.01, *** *p* < 0.001, # *p* < 0.05, ## *p* < 0.01, ### *p* < 0.001).

To further explore the effect of intestinal absorption on plasma concentration and the role of efflux transporters (Mrp2, P-gp) in the absorption, Caco-2 cells were used for the drug uptake assay. The cytotoxicity of Val at different concentrations and MK571 (Mrp2 inhibitor MK571 and P-gp inhibitor Ver) was detected by cell counting kit-8 (cck8) assay (Figure 4A). MK571 (10 μM) and verapamil (100 μM) were selected for subsequent cellular experiments.

The uptake of valsartan in Caco-2 cells increased over time. MK571 significantly enhanced the cellular uptake of valsartan by Caco-2 cells (*p* < 0.05), while verapamil exhibited limited impact on valsartan uptake (Figure 4B). Furthermore, there was a significant difference in the concentration of valsartan between the MK571 and verapamil groups (*p* < 0.05). These findings suggest that Mrp2 plays a pivotal role in mediating the efflux of valsartan, whereas P-gp may not be substantially involved in transporting this compound. In another set of uptake experiments (Figure 4C), the IR group demonstrated a significant reduction in valsartan uptake compared with the control group at 1 h (*p* < 0.05), although there was no significant difference between the IR group and the control group at 2 h. MK571 was capable of reversing the IR-induced decrease in uptake, even causing significantly higher uptake than that of the control group (*p* < 0.05). In both the MK571 group and the IR + MK571 group, IR significantly decreased valsartan uptake at both 1 h and 2 h (*p* < 0.05).

### 3.6. Effect of X-Ray Irradiation on the mRNA Expression of Transporters Related to Valsartan

**Figure 5:**
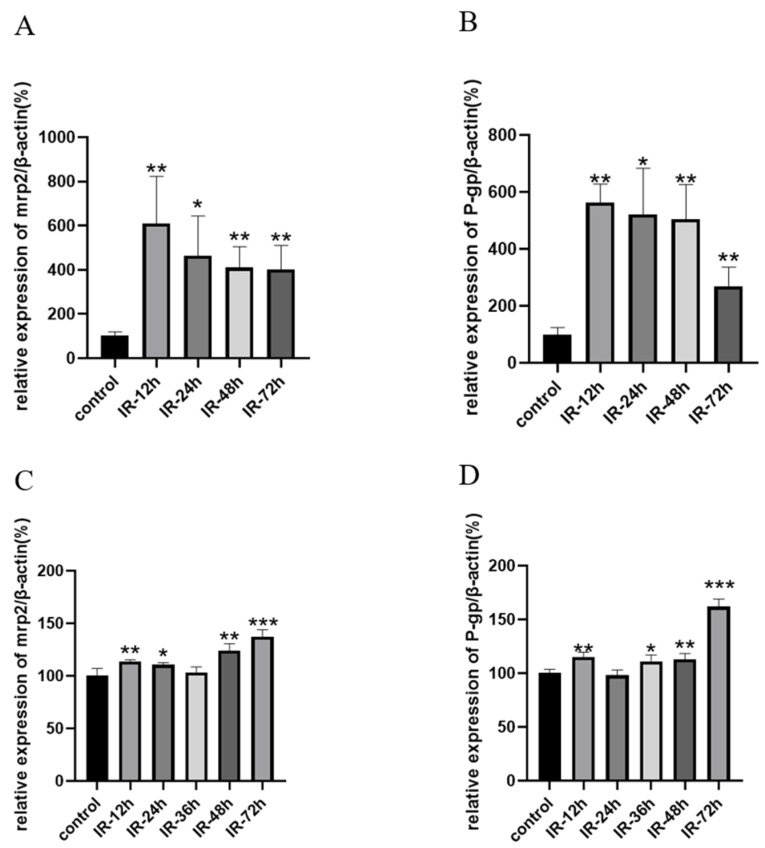
Relative mRNA expression of Mrp2/β-actin (**A**) and P-gp/β-actin (**B**) in rat intestine; Mrp2/β-actin (**C**) and P-gp/β-actin (**D**) in Caco-2 cells after irradiation with 5 Gy of X-rays (Mean ± SD, *n* = 4, * indicates the control group compared, * *p* < 0.05, ** *p* < 0.01, *** *p* < 0.001).

Previous experiments have demonstrated that absorption is the major factor in radiation-induced pharmacokinetic alterations of valsartan. As efflux transporters, Mrp2 and P-gp in the intestine can affect the intestinal absorption of valsartan. The results of RT-qPCR are shown in Figure 5. The expression of Mrp2 and P-gp in the rat intestine increased significantly after radiation (*p* < 0.05), reached the highest level at 12 h after radiation, and then gradually decreased. The expressions of Mrp2 and P-gp in Caco-2 cells were also significantly upregulated after radiation (*p* < 0.05), but the amplitude was much lower than that in rat intestinal tissues. In addition, the liver is also distributed in the abdominal cavity and can also be affected by abdominal irradiation. It is closely related to the secretion and excretion of valsartan into bile, so we detected valsartan-related transporters in the liver. The results were shown in Figure S2. P-gp in the liver maintained the same changes as in intestinal tissue, but Mrp2 and uptake transporter oatp2 in the liver were significantly downregulated after radiation, which indicates that the biological effects of radiation vary from organ to organ.

### 3.7. Detection of Biochemical Indicators of Intestinal Oxidative Stress After Irradiation

**Figure 6:**
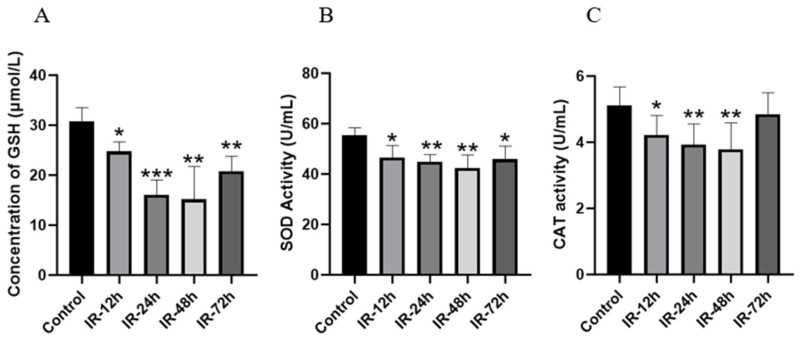
Effect of irradiation with 5 Gy X-ray on concentration of GSH (**A**), SOD activity (**B**), and CAT activity (**C**) in rat jejunum (Mean ± SD, *n* = 4, * indicates the control group compared, * *p* < 0.05, ** *p* < 0.01, *** *p* < 0.001).

GSH is the most significant non-enzymatic antioxidant substance within the body [57], and its level can indirectly indicate the extent of cellular oxidative stress. SOD and CAT are also crucial enzymes for eliminating oxygen free radicals [58]. As shown in Figure 6, compared with the control group, the contents of GSH and the activity of SOD in the jejunal tissue decreased significantly from 12 h to 48 h after IR (*p* < 0.05). Compared with the IR-48 h group, the contents of GSH and the activity of SOD in the IR-72 h group increased but were still lower than those in the control group (*p* < 0.05). In contrast to the control group, CAT activity also declined in a time-dependent manner after IR (*p* < 0.05), reaching the lowest level at 48 h.

### 3.8. The Impact of X-Ray Irradiation on the mRNA Expression of Nrf2 and HO-1

**Figure 7:**
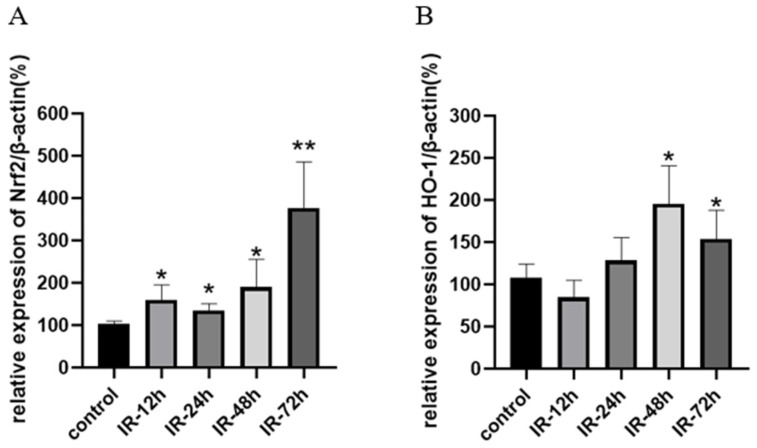
Relative mRNA expression of Nrf2/β-actin (**A**) and HO-1/β-actin (**B**) in rat intestine after irradiation with 5 Gy of X-rays (Mean ± SD, *n* = 4, * indicates the control group compared, * *p* < 0.05, ** *p* < 0.01).

Nrf2 is a key transcription factor that regulates a variety of antioxidant enzymes or substances, including GSH, SOD, and CAT. HO-1 (heme oxygenase-1) is also an antioxidant enzyme regulated downstream of Nrf2 and plays an important role in scavenging ROS produced by oxidative stress [59]. The results of mRNA transcription of Nrf2 as well as HO-1 are shown in Figure 7. Compared with the control group, the mRNA expression of Nrf2 was significantly upregulated after irradiation (*p* < 0.05) and showed a time-dependent manner in general. The expression of HO-1 was significantly increased at 48 h and 72 h after irradiation (*p* < 0.05), which indirectly proves the regulatory role of Nrf2 upregulation on downstream genes.

## 4. Discussion

In order to accurately analyze valsartan, this study first established a quantitative analysis method of valsartan by LC-MS/MS and carried out methodological verification. Telmisartan, a structural analog of valsartan, was selected as the internal standard [60]. Firstly, the ion pairs of valsartan and telmisartan were determined to be *m/z* 436.1→207.1 and *m/z* 515.2→276.1 according to the ion responses in MS_1_ and MS_3_ modes, respectively. The MS parameters and mobile phase composition and ratio were optimized in turn, and the results are described in detail in Section 2.2. According to the guidance principles of “M10: Bioanalytical Method Validation and Sample Analysis”, published by the ICH [56], the selectivity, accuracy, precision, linearity, extraction recovery, and matrix effect of the established analytical method were validated. All the results met the quantitative requirements. The data of method validation are available in the Supplementary Material.

X-rays or γ-rays, as ionizing radiation, can induce molecular dissociation to directly damage macromolecules such as DNA [61], and can also induce the ionization of water molecules to produce reactive oxygen species (ROS) [62]. The ROS generated can also cause damage to macromolecules and lipids in cells, leading to oxidative stress and the activation of various inflammatory factors and apoptosis-inducing factors within cells, and thereby resulting in apoptosis [63]. Consequently, both X-rays and γ-rays are employed in cancer radiotherapy in clinical practice [64], and diverse fractionated doses are utilized in accordance with different radiotherapy modalities. The fractionated dose of conventional standard radiotherapy is 1.8–2 Gy per fraction [65], while high-dose fractionated radiotherapy employs a single dose of more than 2 Gy. Stereotactic body radiation therapy (SBRT) utilizes more than 5 Gy per fraction of radiation dose [66]. In order to simulate the clinical radiation dose and achieve a good effect for the RT-PK study, a dose of 5 Gy and a dose rate of 2 Gy/min were selected.

The in vivo study showed that the pharmacokinetics of valsartan after oral administration was significantly influenced in rats exposed to X-rays in the abdominal region (three finger widths below the xiphoid process) under the cover of a lead mold, as indicated by a 37% reduction in the AUC_0–24h_ and a 52% reduction in the C_max_ of valsartan (Table 1). The occurrence of this phenomenon may be related to reduced absorption, increased metabolism and accelerated excretion. However, valsartan exists in the body in its original form, so metabolic reasons can be ruled out. According to the concentration–time curve (Figure 1) and the calculated pharmacokinetic parameters, this may be related to the decreased intestinal absorption of valsartan after exposure to radiation. However, this inference needs to be verified by further studies.

The excretion of valsartan in urine and feces and the secretion of bile after radiation were examined. As a highly plasma protein-binding drug [67], a small amount of valsartan is excreted in urine. The majority of valsartan (>80%) is absorbed into the liver, secreted into the bile, and ultimately passed into the feces along with the unabsorbed valsartan [68]. Our experimental results (Figure 2) also proved that valsartan is mainly excreted in the feces. The uptake transporters oatps and the efflux transporter Mrp2 in the liver are involved in these processes [40]. Bile secretion results showed that the cumulative excretion fraction of valsartan and the excretion of valsartan in each time period in the bile of irradiated rats decreased (Figure 2A,B), which may be related to the reduction of the total body exposure of valsartan. In addition, our results from the transporter expression study also showed that Mrp2 and oatp1b2 (rat homologue of human OATP1B1/OATP1B3) were downregulated in rat liver (Figure S2), which may also contribute to the reduced bile secretion of valsartan. The opposite trend to the upregulation of intestinal Mrp2 needs attention and further investigation in the future. The absence of a significant change in the fecal excretion of valsartan may be attributed to the low bioavailability of valsartan (23%) [69] and the presence of large amounts of unabsorbed valsartan in feces; however, the decrease in the bile secretion of valsartan counteracts the effect of the increased unabsorbed drug. Although the concentration of valsartan in urine significantly decreased from 4 h to 8 h after irradiation, the total amount was too low to be of practical significance. In conclusion, the results of the study on bile secretion and urinary or fecal excretion of valsartan indicate that accelerated excretion is absent in the RT-PK phenomenon of valsartan. Thus, decreased absorption may have contributed to the decrease in AUC of valsartan in the in vivo study.

To further determine the effect of absorption and the mechanism of the decreased absorption, we designed an ex vivo inverted intestinal sac assay and an uptake assay in Caco-2 cells. Caco-2 is a human clonal colon adenocarcinoma cell line, which is structurally and functionally similar to differentiated small intestinal epithelial cells [70]. Differentiated Caco-2 cells have a microvillous structure and express antibodies such as Mrp2 and P-gp [70]. Caco-2 was often employed to study the mechanism of drug transport and drug absorption. The results of the inverted intestinal sac assay (Figure 3) and the cellular uptake results (Figure 4) confirmed our conjecture that the upregulation of intestinal efflux transporters Mrp2 and P-gp caused by radiation reduced the total amount of drug absorbed into the blood. This hypothesis was supported by our PCR results in the intestine and in Caco-2 cells (Figure 5).

However, based on the results of cellular uptake, Mrp2 seemed to play a more significant role in valsartan transport, while the P-gp inhibitor verapamil did not significantly increase the uptake of valsartan by Caco-2 cells (Figure 4B). We therefore concentrated on intestinal Mrp2 expression as the cause of decreased uptake. Some studies have indicated that oxidative stress within the body can bring about alterations in the expression of metabolic enzymes [71,72] and transporters, which include the Mrp2 [73] and P-gp [35]. To explore whether X-rays also induce an increase in Mrp2 in the rat intestine via oxidative stress, we measured the contents of GSH, SOD, and CAT in the intestinal tissue after radiation (Figure 6), and the results demonstrated that oxidative stress indeed occurred in rats after irradiation.

Nrf2, a crucial intracellular transcription factor that governs oxidative stress [74], is activated in response to oxidative stress [73]. Nrf2 is also implicated in the regulation of multiple transporters [36,37,73], among which Mrp2 is included. Knockdown or silencing of the Nrf2 gene leads to the downregulation of Mrp2 expression [75]. Nrf2 could be regulated at multiple levels [76], encompassing transcriptional regulation [77], post-transcriptional regulation, post-translational regulation [78], and self-stability regulation. For example, knocking down the upstream Nf-κB of Nrf2 can significantly reduce the expression of Nrf2 [75]. Simultaneously, oxidative or covalent modification of cysteine residues Cys273, Cys288, and Cys151 of Keap1 can result in a reduction in Nrf2 ubiquitination, inducing Nrf2 to be released from Keap1 and translocate to the nucleus to exert a transcriptional regulatory role [79]. Nrf2 exerts a positive regulatory effect on various antioxidant factors, including NQO1, HO-1, SOD, CAT, and GSH [80], while also playing a crucial role in GSH regeneration. Our transcriptional analysis revealed significant upregulation of both Nrf2 and its downstream target HO-1 in the rat intestine following radiation exposure (Figure 7). The peak upregulation of Nrf2 occurred at 72 h post-radiation, which was consistent with the observed changes in GSH levels as well as SOD and CAT activities (Figure 6). Simultaneously, the quantity of reactive oxygen species (ROS) rises after radiation exposure, while the expression of Nrf2 gradually escalates, resulting in a gradual reduction in the levels of GSH, SOD, and CAT, but at a declined pace, and a restoration or near-normal level by 72 h post-irradiation. The intestinal expression of Mrp2 mRNA exhibited a similar trend to that of Nrf2, displaying a significant increase following radiation exposure. This finding further supports the regulatory role of Nrf2 in modulating Mrp2 expression through subsequent mechanisms, ultimately leading to reduced absorption of valsartan. The reduction in the peak blood concentration and AUC of valsartan may compromise its antihypertensive efficacy, potentially leading to inadequate blood pressure control and an increased risk of cardiovascular events. Therefore, it is recommended to closely monitor blood pressure fluctuations and promptly adjust the dosage of valsartan in patients with cancer and hypertension undergoing radiotherapy. This will contribute to the sustainability of cancer treatment and avoid the forced discontinuation of cancer drug use due to uncontrolled worsening of hypertension [21].

The biological effects of radiation may vary according to the organ and area exposed. We found that Mrp2 is downregulated in the liver (Figure S2), which may be related to other pathways involved in regulating Mrp2 expression in liver cells [81]. The downregulation of hepatic Mrp2 may result in bile stasis and enhance the hepatotoxicity of specific drugs. Radiation can also upregulate P-gp while downregulating oatp2 (Figure S2). Although p-gp was not shown to be involved in the cellular uptake of valsartan in our experiments, it remains unclear whether P-gp played a role in the reduced intestinal absorption of valsartan following radiation exposure. Given its broad substrate specificity, P-gp-mediated RT-PK interactions can occur with a wide range of drugs and induce toxicity or side effects.

**Figure 8:**
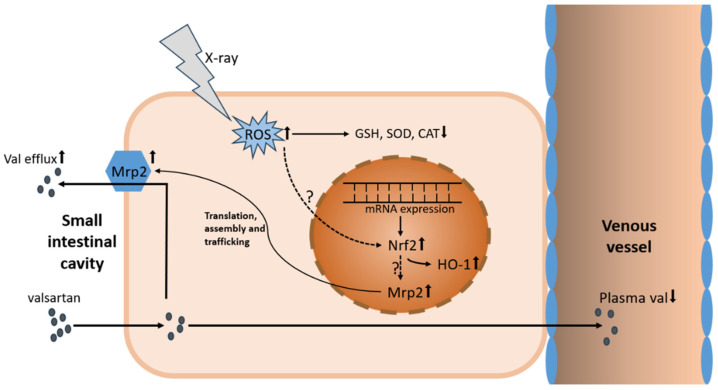
X-ray irradiation induces oxidative stress and upregulates intestinal Nrf2-Mrp2, leading to decreased intestinal absorption of valsartan.

In conclusion, our study has discovered that X-ray irradiation attenuated the intestinal absorption of valsartan, resulting in reduced C_max_ and overall exposure of valsartan in vivo in rats. This phenomenon may be attributed to the upregulation of intestinal Nrf2-Mrp2 through radiation-induced oxidative stress (Figure 8). Further studies are warranted to validate these findings and investigate additional affected transporters, thereby providing a fundamental reference for mitigating potential clinical RT-PK risks in more drugs.

## Figures and Tables

**Table 1:** The pharmacokinetic parameters of valsartan in rat plasma after abdominal irradiation with 5 Gy of X-rays (Mean ± SD, *n* = 6, * indicates the control group compared, * *p* < 0.05, ** *p* < 0.01).

Parameter		Control	IR-5 Gy
t**_1/2_**	h	3.568 ± 0.411	5.032 ± 2.656
C_max_	ng/mL	4428.333 ± 1261.656	2131.667 ± 877.802 **
T_max_	h	0.250 ± 0.000	0.222 ± 0.068
AUC_0–24h_	ng·h/mL	8456.466 ± 1840.136	5269.367 ± 1826.438 *
AUC_0–∞_	ng·h/mL	8813.281 ± 2011.750	5469.390 ± 1791.823 *
V_d_	L/kg	6.254 ± 2.349	14.668 ± 9.456
CL	L/h/kg	1.200 ± 0.352	2.120 ± 1.110
MRT_0–24h_	h	3.839 ± 0.724	4.521 ± 1.631

## Data Availability

The data presented in this study are available on request from the corresponding author.

## Supplementary Materials

The following supporting information can be downloaded at: https://www.mdpi.com/article/10.3390/pharmaceutics17020268/s1, Figure S1: Chromatograms of valsartan and telmisartan in rat blank plasma samples (A,B), LLOQ (C,D), and plasma samples from 0.5 h after drug administration (E,F); Figure S2: Relative mRNA expression of Mrp2/β-actin (A), P-gp/β-actin (B), and oatp1b2/β-actin (C) in rat liver after irradiation with 5 Gy of X-rays (Mean ± SD, *n* = 4, * indicates the control group compared, * *p* < 0.05, ** *p* < 0.01, *** *p* < 0.001); Table S1: System of RT-qPCR; Table S2: RT-qPCR amplification system; Table S3: Sequence of primers used for RT-qPCR; Table S4: Intra-day and inter-day precision and accuracy of the method for the determination of valsartan in rat plasma (Mean, *n* = 6); Table S5: Calibration curve and correlation coefficient of valsartan quantification in rat plasma by LC-MS/MS; Table S6: Matrix effects and extraction recovery of valsartan and telmisartan (IS) in rat plasma (Mean ± SD, *n* = 6).

## Abbreviations

AUC	area under the drug–time curve
ARBs	angiotensin II receptor blockers
BCA	bicinchoninic acid assay
CAT	catalase
cck8	cell counting kit-8
CE	collision energy
CL	clearance rate
C_max_	maximum plasma concentration
CMC-Na	sodium carboxymethylcellulose
CXP	collision cell exit potential
DMEM	Dulbecco’s modified eagle medium
DP	declustering potential
EP	entrance potential
ESI	electrospray ionization
FBS	fetal bovine serum
GSH	glutathione
HO-1	heme oxygenase-1
HQC	high quality control
ICH	International Coordinating Committee for Technical Requirements for Medicinal Products for Human Use
IR	irradiation
IS	internal standard
KRB	Krebs–Ringer Solution
LC-MS/MS	liquid chromatography tandem mass spectrometry
LLOQ	lower limit of quantitation
LQC	lower quality control
MQC	medium quality control
Mrp2	multidrug resistance-associated protein 2
*m/z*	mass-to-charge ratio
Nf-κB	nuclear factor kappa-B
NQO1	NADH quinone oxidoreductase 1
Nrf2	nuclear factor erythroid 2-related factor 2
oatp1b2	organic anion-transporting polypeptide 1b2
OATP1B1/3	organic anion-transporting polypeptide 1B1/3
PBS	phosphate-buffered saline
P-gp	P-glycoprotein
ROS	reactive oxygen species
RSD	relative standard deviation
RT-PK	radiation pharmacokinetics
RT-qPCR	real-time quantification polymerase chain reaction
SBRT	stereotactic body radiation therapy
SOD	superoxide dismutase
t_1/2_	half-life
T_max_	peak time
VAL	valsartan
V_d_	apparent volume of distribution
Ver	verapamil
