# Supplementary material for: X-Ray Irradiation Induces Oxidative Stress and Upregulates Intestinal Nrf2-Mrp2 Pathway, Leading to Decreased Intestinal Absorption of Valsartan
Source: Pharmaceutics. 2025 Feb 17;17(2):268. doi: 10.3390/pharmaceutics17020268 (PMC11860126; doi:10.3390/pharmaceutics17020268)
Supplement: Supplementary file 1 [file pharmaceutics-17-00268-s001.zip › pharmaceutics-3442585-supplementary.pdf]

Supplementary materials

Supplementary figures

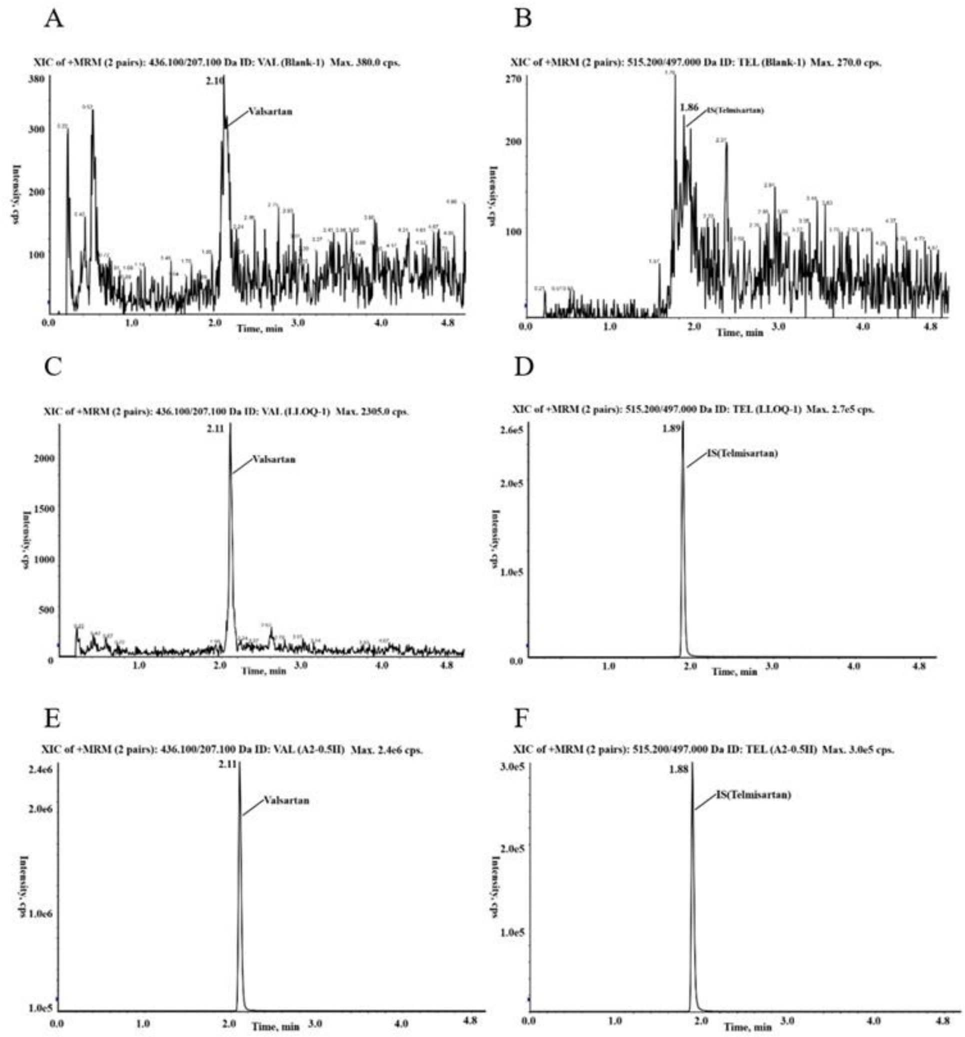

Figure S1 Chromatograms of valsartan and telmisartan in rat blank plasma samples (A, B), LLOQ (C, D), and plasma samples from 0.5 h after drug administration (E, F).

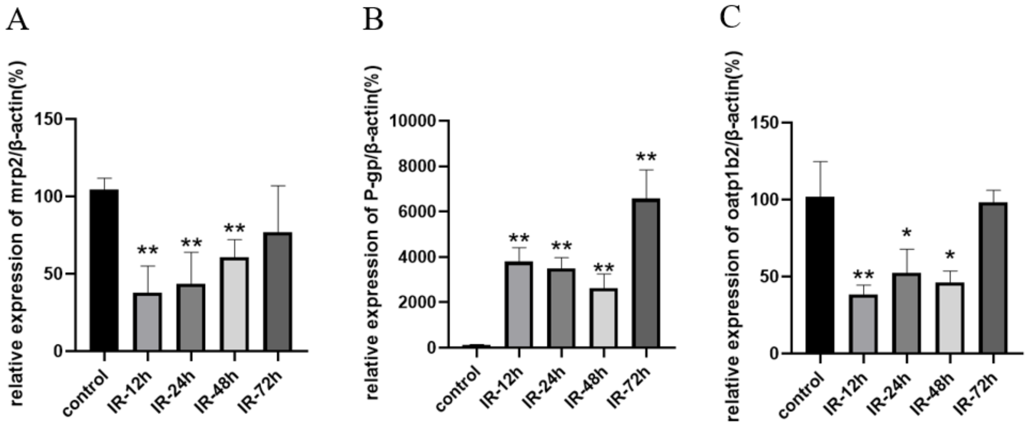

Figure S2 Relative mRNA expression of Mrp2/β-actin (A), P-gp/β-actin (B), oatp1b2/β-actin (C) in rat liver after irradiation with 5Gy of X-rays (Mean ± SD, n=4, *: indicates the control group compared, *p＜0.05, **p＜0.01, ***p＜0.001)

Supplementary tables

Table S1 System of RT-qPCR

reagent	Volume (μL)
TB Green Ex Taq II	10
Primer (F)	0.8
Primer (R)	0.8
cDNA template	1.6
DEPC water	6.8
Total volume	20

Table S2 RT-qPCR amplification system

Steps	Conditions
Step 1	95 ° C for 30s
Step 2 (40 cycles)	95 ° C for 5s; 60 ° C for 30s
Dissolution curve	95 ° C, 10s; 65 ° C, 6s; 95 C, 5s

Table S3 Sequence of primers used for RT-qPCR

Primers	Sequence (5 '-3')
R - beta ACTIN - Forward	CTGAGAGGGAAATCGTGCGTGAC
R - beta ACTIN - Reverse	AGGAAGAGGATGCGGCAGTGG
H - beta ACTIN - Forward	GGCCAACCGCGAGAAGATGAC
H - beta ACTIN - Reverse	GGATAGCACAGCCTGGATAGCAAC
r-ABCC2-Forward	CTCTCGGTCTTATGCGGCGTATTC
r-ABCC2-Reverse	AGACGAAGAACAGGTAGGAGTAGGC
r-MDR1-Forward	CACCGCCTGTCCACCATCC
r-MDR1-Reverse	TGCTCCAGCCTGAACCATCG
r-slco1b2-Forward	TTTGACAGCGTTGCCACATTTCTTC
r-slco1b2-Forward	TCCACTATCTCAGGTGAAGGTCCAG
r-Nrf2-Forward	GCCTTCCTCTGCTGCCATTAGTC
r-Nrf2-Reverse	TGCCTTCAGTGTGCTTCTGGTTG
r-HO-1-Forward	CAGACAGAGTTTCTTCGCCAGAGG
r-HO-1-Reverse	TGTGAGGACCCATCGCAGGAG
h-ABCC2-Forward	GACAACCTCATTCAGACGACCATCC
h-ABCC2-Reverse	GGCTGCCGCACTCTATAATCTTCC
h-MDR1-Forward	TGGCAAAGAAATAAAGCGACTGAATG
h-MDR1-Reverse	TGTTGTCTCCATAGGCAATGTTCTC

Table S4 Intra-day and inter-day precision and accuracy of the method for the determination of valsartan in rat plasma (Mean, n=6)

Compound	Concentration (ng/ml)			Precision (RSD, %)			
	Spiked (ng/ml)	Measured (mean±SD)	Accuracy (%)	Intra-day			Inter-day
				Day1	Day2	Day3	
Valsartan	2	2.01±0.09	100.3	4.84	5.99	2.40	0.71
	5	5.22±0.18	104.4	2.52	4.45	3.08	3.79
	1000	1024.33 ±27.33	102.4	0.78	2.21	1.84	6.22
	3750	3850.56 ±98.01	102.7	2.25	2.26	2.96	2.82

Table S5 Calibration curve and correlation coefficient of valsartan quantification in rat plasma by LC-MS/MS

Compound	Run	Curves	R
Valsartan	1	$\hat{Y}$=0.0025X+0.0094	0.9995
	2	$\hat{Y}$=0.003X-0.003	0.9985
	3	$\hat{Y}$=0.0029X+0.0682	0.9994

Table S6 Matrix effects and extraction recovery of valsartan and telmisartan (IS) in rat plasma (Mean ± SD, n = 6)

Compound	Concentration (ng/ml)	Matrix effect (%)	RSD (%)	Recovery (%)	RSD (%)
Valsartan	5	100.78	7.96	97.41	2.96
	3750	97.78	2.58	103.01	2.60
Telmisartan (IS)	50	103.09	3.24	99.44	2.90
